# Factors and Outcomes Associated with MRCP Use prior to ERCP in Patients at High Risk for Choledocholithiasis

**DOI:** 10.1155/2016/5132052

**Published:** 2016-04-28

**Authors:** Gobind Anand, Yuval A. Patel, Hsin-Chieh Yeh, Mouen A. Khashab, Anne Marie Lennon, Eun Ji Shin, Marcia I. Canto, Patrick I. Okolo, Anthony N. Kalloo, Vikesh K. Singh

**Affiliations:** ^1^Division of Gastroenterology, Department of Medicine, University of California, San Diego, USA; ^2^Division of General Internal Medicine, Department of Medicine, Johns Hopkins University School of Medicine, 1830 E. Monument Street, Baltimore, MD 21205, USA; ^3^Division of Gastroenterology, Department of Medicine, Johns Hopkins University School of Medicine, 2024 E. Monument Street, Room 2-612, Baltimore, MD 21287, USA

## Abstract

*Background*. Consensus guidelines recommend that patients at high risk for choledocholithiasis undergo endoscopic retrograde cholangiopancreatography (ERCP) without additional imaging. This study evaluates factors and outcomes associated with performing magnetic resonance cholangiopancreatography (MRCP) prior to ERCP among patients at high risk for choledocholithiasis.* Methods*. An institutional administrative database was searched using diagnosis codes for choledocholithiasis, cholangitis, and acute pancreatitis and procedure codes for MRCP and ERCP. Patients categorized as high risk for choledocholithiasis were evaluated.* Results*. 224 patients classified as high risk, of whom 176 (79%) underwent ERCP only, while 48 (21%) underwent MRCP prior to ERCP. Patients undergoing MRCP experienced longer time to ERCP (72 hours versus 35 hours, *p* < 0.0001), longer length of stay (8 days versus 6 days, *p* = 0.02), higher hospital charges ($23,488 versus $19,260, *p* = 0.08), and higher radiology charges ($3,385 versus $1,711, *p* < 0.0001). The presence of common bile duct stone(s) on ultrasound was the only independent factor associated with less use of MRCP (OR 0.09, *p* < 0.0001).* Conclusions*. MRCP use prior to ERCP in patients at high risk for choledocholithiasis is common and associated with greater length of hospital stay, higher radiology charges, and a trend towards higher hospital charges.

## 1. Introduction

Gallstone disease is highly prevalent in the United States, affecting 10% of the population and resulting in an annual cost burden of $6.2 billion [1, 2]. Of particular concern is the subset of patients with choledocholithiasis, as potential complications of pancreatitis and cholangitis have mortality rates of 1–3% and about 10%, respectively [3–5]. In 1992, the development of magnetic resonance cholangiopancreatography (MRCP) allowed for highly accurate noninvasive imaging of the bile and pancreatic ducts. Numerous studies have evaluated the efficacy of MRCP for detecting choledocholithiasis, with one large meta-analysis revealing a pooled sensitivity of 92% and specificity of 97% for the detection of stones [6].

Several studies have suggested limiting the use of MRCP to patients at moderate risk for choledocholithiasis and performing endoscopic retrograde cholangiopancreatography (ERCP) in high risk patients [7–10]. Liu et al. evaluated a prospective cohort of patients at high risk of choledocholithiasis (based on clinical, sonographic, and laboratory criteria) prior to laparoscopic cholecystectomy and found a high incidence of common bile duct (CBD) stones in patients with jaundice (71%) and cholangitis (100%), leading the authors to recommend use of ERCP directly in this group. However, the incidence of choledocholithiasis in patients with cholecystitis and pancreatitis was only 29% and 16%, respectively, and the authors recommended MRCP in this intermediate risk group. Other studies, however, have advocated MRCP for both intermediate and high risk groups to minimize the risk of complications from ERCP and potentially decrease hospital costs [11, 12].

Given the uncertainty surrounding the management of choledecholithiasis, the American Society for Gastrointestinal Endoscopy (ASGE) released consensus guidelines for the management of patients with suspected choledocholithiasis in January 2010 [13]. These guidelines classify patients with symptomatic cholelithiasis as high, intermediate, or low risk of choledocholithiasis based on clinical, laboratory (liver biochemical tests), and ultrasound (US) criteria. High risk patients (>50% probability of common bile duct stone) have the presence of a common bile duct (CBD) stone on US, clinical ascending cholangitis, bilirubin > 4 mg/dL, and/or the combination of CBD dilation (>6 mm with an intact gallbladder) with a bilirubin level of 1.8–4 mg/dL. Intermediate risk patients (10–50% probability of CBD stones) have advanced age (>55 years), liver biochemical abnormalities other than bilirubin, and/or clinical suspicion of biliary pancreatitis. Younger patients without bile duct dilation or liver biochemical test abnormalities were considered low risk (<10% probability of CBD stone). As per the guidelines, high risk patients should undergo ERCP without any additional imaging. Intermediate risk patients require further evaluation with endoscopic ultrasound (EUS), MRCP, laparoscopic US, or intraoperative cholangiography (IOC). Low risk patients should proceed directly to cholecystectomy.

While these guidelines were based on available evidence, the proposed algorithm for evaluation of choledocholithiasis has not been validated or studied. This study evaluates a cohort of patients at high risk for choledocholithiasis who all required ERCP to determine frequency of MRCP use and the factors and outcomes associated with MRCP prior to ERCP.

## 2. Methods

An institutional administrative database was searched from 3/2001 to 8/2010 using ICD-9-CM diagnosis codes for choledocholithiasis (574.00–574.9), cholangitis (576.1), and acute pancreatitis (577.0) as well as ICD-9-CM procedure codes for MRCP (52661200) and ERCP (51.85, 51.87, 51.10, 51.19, 51.88, 51.84, and 52.13). All coded diagnoses and procedures were confirmed by reviewing the medical records of each patient. All patients in this cohort underwent ERCP. Patients with a history of cholecystectomy, malignant biliary obstruction, chronic pancreatitis, suspected sphincter of Oddi dysfunction, primary sclerosing cholangitis, HIV cholangiopathy, liver transplantation, chronic liver disease, or sickle cell anemia were excluded. Patients who underwent EUS or were transferred from outside facilities were also excluded. Patients categorized as high risk for choledocholithiasis as per the ASGE guidelines were evaluated [13]. These patients were divided into two groups: (1) patients who underwent MRCP prior to ERCP and (2) patients who underwent ERCP only. High risk patients who only underwent MRCP were not evaluated.

The medical records for each patient were reviewed for the presence of hypotension, defined as mean arterial pressure (MAP) less than 65 mmHg, and the presence of the severe inflammatory response syndrome (SIRS) which was defined as the presence of more than 2 of the following: temperature greater than 38.5 degrees Celsius or less than 35 degrees Celsius, heart rate greater than 90 beats per minute, respiratory rate greater than 20 respirations per minute or PaCO_2_ < 35 mmHg, and white blood cell count greater than 12,000/cu mm or less than 4000/cu mm or greater than 10% band cells [14]. Persistent SIRS was defined as SIRS lasting >48 hours. Time to MRCP was defined as the time between US and MRCP (in hours). Time to ERCP was defined as the time between US and ERCP (in hours). The incidence of post-ERCP pancreatitis was compared between the two groups and defined as the presence of changes consistent with acute pancreatitis on abdominal computed tomography (CT) scans. Follow-up was performed until 8/2011 to assess for surgical intervention, recurrent choledocholithiasis, and need for further ERCPs. Comorbidity was assessed using the Charlson comorbidity index [15].

### 2.1. Statistical Analysis

Patient demographics, clinical, laboratory, radiologic, and endoscopic data, as well as charges, were compared between the groups using chi-square test for proportions, Student's *t*-test for means, and Wilcoxon rank-sum test for medians. Demographic, laboratory, radiologic, and patient data were evaluated using multivariable logistic regression to identify predictors of MRCP use. All analyses were performed using SAS version 9.2 (Cary, NC).

## 3. Results

A total of 2,313 patients were evaluated with 224 patients being classified as high risk for choledocholithiasis. One hundred and seventy-six patients (79%) underwent ERCP only, while 48 patients (21%) underwent MRCP prior to ERCP.  Table 1 displays the comparison of demographic and clinical characteristics between patients who underwent MRCP prior to ERCP and patients who only had ERCP performed. There was no difference in age and gender between these two groups. Both groups had similar rates of SIRS, hypotension, need for ICU, blood stream infections, and mortality. Charlson comorbidity scores were also similar between the two groups. Patients that underwent MRCP and ERCP experienced a significant delay in time to ERCP compared to patients that underwent ERCP only (72.5 hours versus 35 hours, *p* < 0.0001). The delay in ERCP was associated with a significantly longer median length of stay (8 days versus 6 days, *p* = 0.02) and higher median radiology charges ($3,385 versus $1,711, *p* < 0.0001). There was a trend towards higher median total hospital charges for patients who underwent both MRCP and ERCP ($23,488 versus $19,260, *p* = 0.08). The majority of patients were admitted to medical services (approximately two-thirds), a ratio that was similar in both groups.

Table 2 compares the procedural characteristics and complications between patients who underwent MRCP prior to ERCP and those patients who only underwent ERCP. Procedural characteristics including sphincterotomy, stone removal, sludge removal, and stent placement were not significantly different between both groups. There was no difference in number of ERCPs or unsuccessful ERCPs between the two groups. Post-ERCP complications including post-ERCP pancreatitis and duodenal perforation were also similar between the groups. There were no instances of postsphincterotomy bleeding requiring blood transfusion in either group. Of the patients that underwent MRCP prior to ERCP, 54% (26/48) were found to have choledocholithasis on MRCP, 42% (20/48) had normal MRCPs, and 4% (2/48) demonstrated strictures. Of the 20 patients with normal MRCPs, all underwent subsequent ERCP and 25% (5/20) were found to have stones on ERCP, while 20% (4/20) were found to have sludge.

Table 3 demonstrates the multivariable analysis of factors which influence the use of MRCP in patients at high risk of choledocholithasis. The presence of CBD stone on US (OR 0.09, 95% CI (0.03, 0.28), *p* < 0.0001) was associated with a 91% reduction in use of MRCP. The remainder of the factors, including those recommended by the ASGE guidelines, such as ascending cholangitis, bilirubin greater than 4, and CBD dilatation with bilirubin 1.8–4 mg/dL, were not statistically significant in the adjusted analysis. Patient and clinical factors such as age, hypotension, and ICU status also did not influence the use of MRCP prior to ERCP.

With regard to surgical characteristics, there was no difference in the proportion of patients who underwent inpatient cholecystectomies in the two groups. Postdischarge cholecystectomies were also similar between the two groups and there were no significant differences in patient follow-up, incidence of recurrent choledocholithiasis, or total number of ERCPs between the two groups.

## 4. Discussion

Our study evaluated a cohort of patients at high risk for choledocholithiasis who all required ERCP to determine frequency of MRCP use and the factors and outcomes associated with MRCP prior to ERCP. There are several important findings from this study. The first is that MRCP use is quite common among patients at high risk of choledocholithiasis and is associated with increased cost and length of stay but does not influence patient or procedural outcomes. The second is that the decision to perform MRCP is influenced primarily by the absence of CBD stones on US imaging.

Our retrospective study evaluated a cohort of patients at high risk of choledocholithiasis who all underwent subsequent ERCP. Within this group, nearly 1 in 5 patients also underwent initial MRCP prior to ERCP. The utilization of MRCP in this high risk population is associated with greater length of hospital stay, delay in time to ERCP, higher radiology charges, and a trend towards higher hospital charges. Interestingly, measures reflecting the severity of preprocedure clinical status such as the requirement of ICU stay, blood culture positivity, number of SIRS criteria, persistent SIRS, Charlson comorbidity index, and hypotension were similar between both groups. In addition, the frequency of admission to medical versus surgical services was also similar between both groups, suggesting the utilization of MRCP in this setting is common among both surgeons and internists.

There have been multiple prior studies which have evaluated the use of MRCP in suspected choledocholithiasis. Many authors have advocated and investigated the use of an algorithmic approach to managing choledocholithasis based on risk stratification and selective use of MRCP and ERCP [8, 16]. This risk stratified algorithm is similar to the approach endorsed by the most recent ASGE guidelines. Two studies recommended performing MRCP in high risk patients. In a study by Kim et al., 70% of the patients in the high risk group were found to have CBD stones but the authors suggested that MRCP should be performed in all risk groups to reduce the risks of ERCP [12]. Similarly, Demartines et al. recommended use of MRCP in high risk patients to minimize diagnostic ERCPs and to reduce overall healthcare costs [11]. However, the present study demonstrates that ERCP was performed in a large number of high risk patients despite having normal MRCPs. In addition, charges were significantly higher in the group of patients who underwent both MRCP and ERCP.

In terms of clinical outcomes, patients undergoing MRCP had no significant difference in mortality compared to patients undergoing ERCP only. Procedural characteristics and complications were also similar between both groups. An interesting finding was that the 20 patients (42% of MRCP group) who had normal MRCP underwent ERCP, which further raised the question of the utility of obtaining an MRCP if the decision to perform an ERCP will not be altered by a normal MRCP result. Other studies have also demonstrated that MRCP results do not significantly impact the decision to perform ERCP. Sahai et al. conducted a prospective assessment of the ability of MRCP to obviate ERCP in patients with a variety of pancreaticobiliary disorders and found that MRCP would prevent less than 3% of ERCPs [9]. One possible issue with this study is that it was performed in 1997 and physician confidence in MRCP may have been limited, given the nascent nature of the technology. Regardless, our findings demonstrate that, in high risk patients who have a high pretest probability of choledocholithasis, normal MRCP results often do not result in prevention of subsequent ERCP.

Among the four ASGE criteria that establish patients as high risk for choledocholithiasis, only the finding of a CBD stone on US consistently directed patients to ERCP over obtaining initial MRCP. Clinicians appear to heavily weigh biliary stone(s) visualized on US as the primary impetus to proceed to ERCP. Surprisingly, clinical factors such as age, hypotension, ICU status, and comorbidities were not predictors of MRCP use in the adjusted analysis. A prior meta-analysis demonstrated similar findings that no single predictor is completely accurate in predicting CBD stones prior to cholecystectomy but that CBD stone seen on US is the most reliable predictor for choledocholithiasis on subsequent ERCP or at surgery [17, 18]. The other predictors of choledocholithiasis were cholangitis, bilirubin > 1.7 mg/dL, and CBD dilatation [17, 18]. Further emphasis on the other risk factors for choledocholithiasis may help reduce the overutilization of MRCP in patients at high risk of choledocholithiasis.

There are several limitations to this study. The first is the retrospective nature that relies on the accuracy of ICD-9 coding. However, every chart was individually reviewed to confirm all diagnoses and procedures. The second limitation is the potential generalizability of the results in nontertiary hospital setting. Choledocholithiasis is commonly managed in community hospitals and while the availability of ERCP at community hospitals has increased greatly over time, it is possible that ERCP may not be as readily available, resulting in increased use of MRCP. The third limitation is our study evaluated charges as opposed to costs. Charges are often influenced by a variety of external factors and may not be as accurate of a measure of true cost. In addition, Maryland operates under a Medicare waiver; therefore, the state sets payment rates to hospitals and providers. This results in charge data that may be significantly different compared to other states.

In conclusion, our study showed that around 1 in 5 patients that are at high risk for choledocholithiasis undergo MRCP prior to ERCP contrary to the ASGE consensus guidelines. The use of MRCP in this high risk group is associated with increased cost and length of stay but does not significantly influence patient or procedural outcomes. Given these findings, better practice adherence to high risk stratification guidelines would result in systemic cost savings without compromising patient outcomes.

## Figures and Tables

**Table 1:** Demographic, clinical, and cost characteristics of patients at high risk of choledocholithiasis.

	MRCP + ERCP (*n* = 48)	ERCP only (*n* = 176)	*p* value
Age, mean (SD)	53.7 (19.2)	55.4 (19.4)	0.58
Female	60.4%	57.9%	0.76
SIRS criteria			
0	11 (22.9%)	59 (33.5%)	0.28
1	18 (37.5%)	40 (22.7%)	
2	11 (22.9%)	36 (20.5%)	
3	6 (12.5%)	35 (19.9%)	
4	2 (4.2%)	6 (3.4%)	
>2 SIRS criteria	19 (39.6%)	77 (43.6%)	0.63
Persistent	7 (14.6%)	18 (10.2%)	0.23
MAP < 65	5 (10.4%)	13 (7.4%)	0.70
Need for ICU	7 (14.6%)	40 (22.7%)	0.22
Positive blood cultures	7 (14.6%)	27 (15.3%)	0.9
Mortality	2 (4.2%)	9 (5.1%)	0.91
Charlson comorbidity index, median [*Q*_1_, *Q*_3_]	2 [0, 5]	2 [0, 4]	0.87
Time to MRCP (hrs), median [*Q*_1_, *Q*_3_]	24 [10, 42.8]		
Time to ERCP (hrs), median [*Q*_1_, *Q*_3_]	72.5 [43, 119]	35 [13, 50]	<0.0001
LOS (days), median [*Q*_1_, *Q*_3_]	8 [6, 15]	6 [4, 10]	0.02
Total hospital charges ($), median [*Q*_1_, *Q*_3_]	23488 [15707, 35820]	19260 [10860, 32502]	0.08
Radiology charges ($), median [*Q*_1_, *Q*_3_]	3385 [2261–6058]	1711 [1018–3272]	<0.0001
Primary service of admission			
Medicine	32 (66.7%)	114 (64.8%)	
Surgery	16 (33.3%)	62 (35.2%)	0.807

MAP: mean arterial pressure.

**Table 2:** Procedural characteristics and complications of patients at high risk of choledocholithiasis.

	MRCP + ERCP (*n* = 48)	ERCP only (*n* = 176)	*p* value
Number of ERCPs			
1	43 (89.6%)	154 (87.5%)	0.91
2	4 (8.3%)	19 (10.8%)	
>3	1 (2.1%)	3 (1.7%)	
Sphincterotomy	43 (89.6%)	145 (82.4%)	0.23
Stone removal	24 (50.0%)	109 (61.9%)	0.18
Sludge removal	19 (39.6%)	71 (40.3%)	0.92
Stent placement	27 (56.3%)	81 (46.0%)	0.21
Unsuccessful ERCP	3 (6.3%)	14 (8.1%)	1
Post-ERCP pancreatitis	2 (4.2%)	4 (2.3%)	0.61
Bleeding (requiring blood transfusion)	0	0	NA
Perforation	1 (2.1%)	1 (0.6%)	0.38

LOS: length of stay.

**Table 3:** Multivariable analysis of predictors of MRCP use in patients at high risk of choledocholithiasis.

Variable	Odd ratio	95% CI	*p* value
Ascending cholangitis	0.72	(0.31, 1.7)	0.45
Bilirubin > 4	0.84	(0.26, 2.7)	0.77
CBD stone on US	0.09	(0.03, 0.28)	<0.0001
CBD dilatation and bilirubin 1.8–4	1.05	(0.30, 3.65)	0.93
Charlson comorbidity score	1.07	(0.86, 1.32)	0.53
Age	0.99	(0.97, 1.02)	0.61
Map < 65	1.08	(0.33, 3.56)	0.9
ICU status (ref. = ICU patient)	0.49	(0.19, 1.29)	0.15

## Abbreviations

US: Ultrasound
EUS: Endoscopic ultrasound
MRCP: Magnetic resonance cholangiopancreatography
IOC: Intraoperative cholangiogram
ERCP: Endoscopic retrograde cholangiopancreatography
ASGE: American Society for Gastrointestinal Endoscopy
CBD: Common bile duct
MAP: Mean arterial pressure
SIRS: Severe inflammatory response syndrome
CT: Computed tomography.

## References

[1] Kummerow K. L., Shelton J. M., Phillips S. (2012). Predicting complicated choledocholithiasis. *Journal of Surgical Research*.

[2] Everhart J. E., Ruhl C. E. (2009). Burden of digestive diseases in the United States part I: overall and upper gastrointestinal diseases. *Gastroenterology*.

[3] Attasaranya S., Fogel E. L., Lehman G. A. (2008). Choledocholithiasis, ascending cholangitis, and gallstone pancreatitis. *Medical Clinics of North America*.

[4] Lee J. G. (2009). Diagnosis and management of acute cholangitis. *Nature Reviews Gastroenterology and Hepatology*.

[5] Armstrong C. P., Taylor T. V., Jeacock J., Lucas S. (1985). The biliary tract in patients with acute gallstone pancreatitis. *British Journal of Surgery*.

[6] Romagnuolo J., Bardou M., Rahme E., Joseph L., Reinhold C., Barkun A. N. (2003). Magnetic resonance cholangiopancreatography: a meta-analysis of test performance in suspected biliary disease. *Annals of Internal Medicine*.

[7] Liu T. H., Consorti E. T., Kawashima A. (1999). The efficacy of magnetic resonance cholangiography for the evaluation of patients with suspected choledocholithiasis before laparoscopic cholecystectomy. *The American Journal of Surgery*.

[8] Calvo M. M., Bujanda L., Calderón A. (2002). Role of magnetic resonance cholangiopancreatography in patients with suspected choledocholithiasis. *Mayo Clinic Proceedings*.

[9] Sahai A. V., Devonshire D., Yeoh K. G. (2001). The decision-making value of magnetic resonance cholangiopancreatography in patients seen in a referral center for suspected biliary and pancreatic disease. *American Journal of Gastroenterology*.

[10] Dwerryhouse S. J., Brown E., Vipond M. N. (1998). Prospective evaluation of Magnetic Resonance Cholangiography to detect common bile duct stones before laparoscopic cholecystectomy. *British Journal of Surgery*.

[11] Demartines N., Eisner L., Schnabel K., Fried R., Zuber M., Harder F. (2000). Evaluation of magnetic resonance cholangiography in the management of bile duct stones. *Archives of Surgery*.

[12] Kim J. H., Kim M.-J., Park S. I. (2002). MR cholangiography in symptomatic gallstones: diagnostic accuracy according to clinical risk group. *Radiology*.

[13] Maple J. T., Ben-Menachem T., Anderson M. A. (2010). The role of endoscopy in the evaluation of suspected choledocholithiasis. *Gastrointestinal Endoscopy*.

[14] Bone R. C., Balk R. A., Cerra F. B. (1992). Definitions for sepsis and organ failure and guidelines for the use of innovative therapies in sepsis. *Chest*.

[15] Charlson M. E., Pompei P., Ales K. L., MacKenzie C. R. (1987). A new method of classifying prognostic comorbidity in longitudinal studies: development and validation. *Journal of Chronic Diseases*.

[16] Liu T. H., Consorti E. T., Kawashima A. (2001). Patient evaluation and management with selective use of magnetic resonance cholangiography and endoscopic retrograde cholangiopancreatography before laparoscopic cholecystectomy. *Annals of Surgery*.

[17] Abboud P.-A. C., Malet P. F., Berlin J. A. (1996). Predictors of common bile duct stones prior to cholecystectomy: a meta-analysis. *Gastrointestinal Endoscopy*.

[18] Barkun A. N., Barkun J. S., Fried G. M. (1994). Useful predictors of bile duct stones in patients undergoing laparoscopic cholecystectomy. *Annals of Surgery*.

